# Concurrent inflammatory, hemorheological and macrovascular responses to a 230-km ultramarathon: an exploratory study

**DOI:** 10.1038/s41598-026-55821-1

**Published:** 2026-06-01

**Authors:** Marijke Grau, Jonas Bruns, Lucas John, Moritz Munk, Michael Siebers, Wilhelm Bloch, Daniel A. Bizjak

**Affiliations:** 1 Molecular and Cellular Sports Medicine, Institute of Cardiovascular Research and Sports Medicine, German Sport University Cologne, 50933 Cologne, Germany; 2 Department of Internal Medicine, Division of Sports and Rehabilitation Medicine, University Hospital Ulm, 89075 Ulm, Germany; 3 Center for Translational Neuro- and Behavioral Sciences, Institute of Forensic Psychiatry and Sex Research, University of Duisburg-Essen, 45130 Essen, Germany

**Keywords:** Ultramarathon, Inflammatory marker, Red blood cell aggregation, Nitric oxide, Pulse wave velocity, Blood pressure, Biomarkers, Cardiology, Diseases, Medical research, Physiology

## Abstract

To investigate the concurrent physiological response to extreme endurance exercise by examining inflammatory, hemorheological, endothelial, and vascular adaptations following an ultramarathon. Twelve runners (9 men/3 women; 48 ± 7 years) participating in a 230-km non-stop ultramarathon were assessed before and immediately after the race. Systemic inflammatory and oxidative stress markers, indices of red blood cell (RBC) aggregation and fibrinogen, plasma nitrite as a marker of nitric oxide (NO) bioavailability, and macrovascular hemodynamic parameters were measured. White blood cell count (WBC) (*p* < 0.001), interleukin (IL)-6 (*p* = 0.0002), IL-10 (*p* = 0.0002) and C-reactive protein (CRP) (*p* < 0.001) increased, while plasma free reactive oxygen species (ROS) (*p* = 0.0043) and total antioxidant capacity (*p* = 0.0041) decreased post-race. RBC aggregation increased (*p* = 0.0003) in concert with elevated fibrinogen (*p* < 0.0001). Plasma nitrite increased post-race (*p* = 0.0013). Macrovascular hemodynamics exhibited increased heart rate (*p* < 0.0001) with preserved pulse wave velocity (PWV; *p* = 0.257) and central pressures. Wave reflection indices were altered, with reduced augmentation index (AIx; *p* = 0.034), whereas heart rate–standardized AIx75 remained unchanged (*p* = 0.104), alongside an increase in diastolic reflection area (DRA; *p* = 0.020) and divergent peripheral pressure responses. The ultramarathon was associated with pronounced inflammatory responses accompanied by increased fibrinogen-related RBC aggregation and elevated plasma nitrite concentrations, while macrovascular properties remained largely preserved. Together, these findings suggest that acute responses to extreme endurance exercise involve parallel inflammatory, hemorheological, endothelial, and macrovascular alterations, with cardiovascular adjustments primarily reflecting functional changes in peripheral vascular regulation rather than substantial changes in central arterial mechanical properties.

## Introduction

Ultra-endurance running represents an extreme physiological stressor in which prolonged mechanical loading, sustained sympathetic activation, energy deficit, dehydration, and sleep deprivation occur simultaneously. These stressors can acutely perturb vascular function, hemostasis, and systemic inflammation – systems that are biologically intertwined, yet are often investigated separately in ultramarathon field studies^1–4^.

A prominent hallmark of ultra-endurance exercise is a marked inflammatory response. Ultramarathon running elicits robust increases in interleukin (IL)-6, IL-10, and acute-phase proteins such as C-reactive protein (CRP) in the immediate post-race period, reflecting a coordinated systemic immune activation^1^. IL-6 plays a central mechanistic role in the acute-phase cascade by stimulating hepatic synthesis of CRP^1,3^ and fibrinogen^5^, thus linking cytokine signaling to downstream changes in blood proteins that may influence blood viscosity, clot formation, and vascular biology.

In parallel, prolonged endurance exercise can transiently shift hemostatic balance. Schobersberger and colleagues^6^ reported alterations in coagulation markers following a long-distance ultramarathon, including increased fibrinogen concentrations and concurrent changes in pro- and anticoagulant activity linked to systemic inflammatory responses. In this context, the net hemostatic profile depends on race characteristics, environmental stressors, hydration status, and timing of assessment. Importantly, fibrinogen is a key determinant of red blood cell (RBC) aggregation, suggesting a plausible mechanistic link between exercise-induced inflammatory–hemostatic activation and potential changes in RBC aggregation. RBC aggregation, defined as the reversible formation of rouleaux and larger RBC clusters at low shear rates, alters blood rheology and can impair microvascular perfusion while also influencing peripheral resistance and hemodynamics in the microcirculation^7^. However, direct evidence for post-ultramarathon alterations in RBC aggregation remains scarce, and existing data are limited to highly specific race conditions^8^, precluding robust conclusions about isolated effects of prolonged endurance load per se. Consequently, it remains unclear to what extent hemostatic and inflammatory perturbations translate into meaningful changes in RBC aggregation during a non-stop ultramarathon without additional environmental conditions such as altitude exposure. Beyond their hematological relevance, RBC aggregation may also affect vascular function and central hemodynamics^7,9^. Accordingly, vascular parameters such as arterial stiffness, typically assessed by PWV, and indices of pulse wave reflection have received increasing attention in the context of prolonged endurance exercise^10^. However, previous studies investigating macrovascular responses to ultramarathon running have reported heterogeneous findings. While some investigations have observed stable carotid–femoral pulse wave velocity (cf-PWV) despite substantial physiological stress^11^, increases in PWV have also been described in certain race settings^12^, suggesting that the macrovascular response may depend on factors such as race duration, environmental stress, hydration status, and measurement timing. Beyond arterial stiffness, ultramarathon studies have also reported exercise-induced alterations in central hemodynamics and wave reflection indices, although these responses appear to vary considerably between studies and race conditions^13,14^. Importantly, indices derived from pulse wave analysis, such as augmentation index, are influenced not only by arterial properties but also by heart rate, autonomic activity, and peripheral vascular tone. Because these physiological factors are markedly perturbed immediately after ultra-endurance exercise, interpretation of post-race vascular measurements remains challenging^15,16^.

Although these bodies of literature are substantial, a key gap remains: to our knowledge, no study has examined the parallel interplay between inflammatory activation, microvascular hemorheology and hemostasis, and macrovascular arterial hemodynamics within the same athletes across matched peri-race time points. Previous work from our group in the same race cohort has already addressed hematological RBC and hemorheological parameters, but primarily focused on RBC deformability and intracellular RBC-related nitric oxide signaling mechanisms^17^. The present study extends these observations toward a multidimensional vascular–hemorheological perspective by investigating RBC aggregation and fibrinogen in relation to inflammatory activation, plasma nitrite bioavailability, arterial stiffness, wave reflection, and systemic hemodynamics following a 230-km non-stop ultramarathon. Therefore, the aim of the present exploratory pilot study was to characterize the acute responses to a 230-km non-stop ultramarathon across interrelated inflammatory, microvascular, and macrovascular domains, including (i) systemic inflammatory responses (IL-6, IL-10, CRP, and white blood cell (WBC) count, plasma free reactive oxygen species (ROS), plasma total antioxidant capacity (TAC)), (ii) microcirculatory markers (RBC aggregation and hemostatic factors such as fibrinogen), (iii) endothelial and vasoactive signaling reflected by plasma nitrite (NO_2_^−^) as a marker of systemic nitric oxide (NO) bioavailability, and (iv) macrovascular function and central hemodynamics (PWV, blood pressure, wave reflection/augmentation index, heart rate).

## Methods

### Participation, race conditions, and sample collection

The TorTour de Ruhr^®^ is an invitation-only 230-km non-stop ultramarathon requiring extensive prior ultra-endurance experience and medical clearance. Participants provided written informed consent, and the study was approved by the Ethics Committee of the German Sport University Cologne in accordance with the Declaration of Helsinki (12/2024). As the race conditions have been described previously, only the most relevant information is summarized here^17^. Twelve (3 female/9 male) runners completed the race and were included in the final analyses, with a mean completion time of 32 h 52 min 34 s (± 3 h 34 min 54 s). Basic anthropometric data were: age (48.3 ± 6.6 years), height (177.6 ± 8.0 cm), weight (71.2 ± 13.7 kg), with no significant weight changes post-race. Vascular parameters and venous blood samples were obtained pre-race (May 17, 2024, 16:00–20:00 h) and within 10 min post-race (May 19, 2024). The immediate post-race assessment was intentionally chosen to capture acute physiological responses to the ultramarathon before rapidly changing parameters, particularly plasma nitrite and hemodynamic indices, returned toward baseline. Blood samples were processed and stored immediately for subsequent analyses. Race conditions comprised moderate temperatures with intermittent rain and variable humidity^3,17^.

### Sample preparation

A complete blood count was performed at a medical laboratory (Dr. Wisplinghoff, Cologne, Germany). Only the WBC data were used for the present study; the overall results have already been published^17^.

For plasma analysis, EDTA (K2E, EDTA vacutainer, BD, Heidelberg, Germany) anticoagulated whole blood was separated by centrifugation at 1000 × g for 15 min for IL-6, IL-10, CRP, TAC, fibrinogen, NO_2_^−^ measurements and further separated at 10,000 × g for 5 min for ROS measurements. The plasma was stored at − 80 °C until analysis.

### Sample analysis

#### Fibrinogen, IL-6, IL-10, CRP measurements

All assays and measurements were conducted in accordance with the manufacturers’ protocols. For fibrinogen measurements, the Competition ELISA Kit for Fibrinogen (antibodies-online GmbH, Aachen, Germany) was used in which sample fibrinogen competes with immobilized antigen for antibody binding and was detected colorimetrically at 450 nm (Multiskan FC Photometer, Thermo Fisher Scientific, Waltham, USA). Plasma concentrations of IL-6, IL-10 and CRP were quantified using Quantikine^®^ Human IL-6, IL-10 and CRP ELISA kits (R&D Systems, Minneapolis, Minnesota, USA), which are solid-phase sandwich immunoassays employing specific capture and detection antibodies and colorimetric detection (450 nm). The assays represent quantitative measurements based on standard curves generated with recombinant human analytes.

#### Parameters of oxidative stress: free ROS/RNS levels and total antioxidant capacity

Plasma free radical content was measured using the OxiSelect In Vitro ROS/RNS Assay Kit (Cell Biolabs Inc., San Diego, USA), a fluorescence-based assay in which reactive oxygen and nitrogen species generate a fluorogenic signal measured at 480 nm excitation and 530 nm emission (Fluoroskan Ascent Microplate Fluorometer; Thermo Fisher Scientific, Waltham, USA).

TAC was assessed using the Total Antioxidant Capacity Assay Kit (Abcam, Cambridge, UK), a colorimetric assay in which antioxidants in the sample reduce a copper(II)–to–copper(I) reagent, producing a chromogen measured spectrophotometrically at 570 nm according to the manufacturer’s protocol. Total antioxidant capacity was expressed as Trolox equivalent capacity, following the principle that the sample´s antioxidant capacity is compared to that of Trolox.

#### Nitrite measurement

Plasma nitrite concentration was measured using an ozone-based chemiluminescence NO detector (CLD 88e, EcoPhysics AG, Duernten, Switzerland) as previously described^18,19^. Briefly, plasma samples were thawed on ice and injected into an acidified tri-iodide solution, which quantitatively reduces nitrite to NO gas, subsequently detected via gas-phase chemiluminescence following reaction with ozone. Data were acquired and analyzed using the Chart FIA software (eDAQ Chart v.5.5.11; eDAQ Pty Ltd., Australia) by integrating the area under the curve. Nitrite concentrations were calculated from standard curves generated using sodium nitrite standards (sodium nitrite, Merck KGaA, Darmstadt, Germany) prepared in 1 x phosphate-buffered saline (pH 7.4). All samples were measured in triplicate, and measurements were performed under identical experimental conditions.

### Measurement of macrovascular parameters

Macrovascular parameters were assessed non-invasively using an oscillometric, upper-arm cuff–based device (Arteriograph, TensioMed Ltd., Budapest, Hungary). After a standardized 15-min seated rest period, the cuff was placed on the upper arm approximately two finger widths above the antecubital fossa and inflated above systolic blood pressure to transiently occlude the brachial artery, allowing recording of the pulse pressure waveform and reflected pressure waves. The Arteriograph uses these suprasystolic oscillometric signals obtained at the upper-arm cuff to estimate central/aortic arterial stiffness and aortic pulse wave velocity. Central hemodynamic and arterial stiffness parameters were derived from the oscillometric signal using the manufacturer’s validated algorithm^20–22^, including PWV, brachial and central blood pressure, augmentation index (AIx), and wave reflection indices such as DRA. DRA is a complex dimensionless parameter that reflects the intensity and duration of diastolic wave reflection. The higher the DRA, the better the perfusion of the left coronary artery. AIx describes the peripheral resistance of arteries and arterioles and represents the impact of the reflected wave on central systolic blood pressure. Augmentation index was normalized to a heart rate of 75 bpm (AIx75) using the established linear heart rate correction to reduce heart rate–dependent variability in wave reflection measures^23^. Measurements were performed under standardized conditions, and the mean of valid recordings was used for subsequent analyses in accordance with current recommendations for non-invasive vascular assessment^24,25^.

### Statistics

Sample analysis was performed in random order with investigators blinded to experimental condition. Statistical analysis and figure preparation were performed using GraphPad Prism (GraphPad Prism 9.5.1; San Diego, CA, USA). Data normality was assessed with the Shapiro-Wilk test. Pre- to post-race comparisons were conducted using two-tailed paired t-test for normally distributed variables or Wilcoxon matched-paired signed rank test when normality was not met. Associations between exercise-induced changes (Δ) in fibrinogen concentration and RBC aggregation index, IL-6 and fibrinogen and plasma nitrite and AIx brachial were assessed using Spearman’s rank correlation coefficient. Δ values were calculated as post-race minus pre-race values. Data are reported as mean ± standard deviation and as individual pre–post changes. Statistical significance was set at *p* ≤ 0.05.

## Results

### Inflammatory and redox markers

**Fig. 1:**
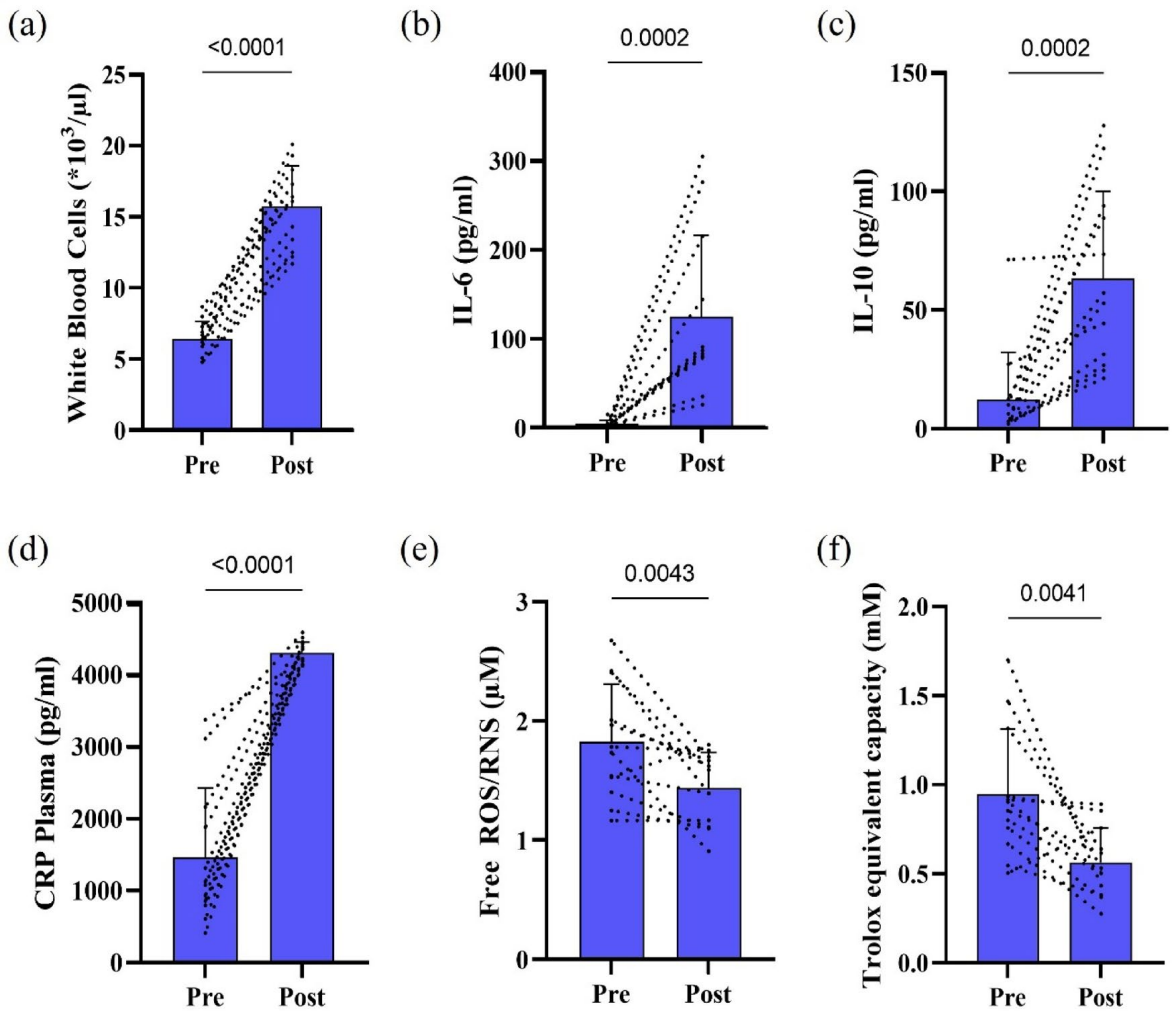
Peri-race changes in inflammatory and redox markers following an ultramarathon. (**A**) WBC, (**B**) IL-6, (**C**) IL-10, and (**D**) CRP increased significantly from pre- to post-race, indicating pronounced systemic inflammatory activation. In contrast, (**E**) plasma ROS and (**F**) plasma TAC decreased significantly post-race, reflecting an acute depletion of circulating antioxidative buffering capacity in the immediate post-race period. Exact p-values are reported within each panel. Individual pre–post trajectories are shown as dashed lines, with bars representing mean ± SD (*n* = 12).

The ultramarathon elicited a pronounced systemic inflammatory response, as evidenced by significant post-race increases in WBC count (*p* < 0.0001; Fig. 1A) and the cytokines IL-6 (*p* = 0.0002; Fig. 1B) and IL-10 (*p* = 0.0002, Fig. 1C), alongside elevated CRP concentrations (*p* < 0.0001; Fig. 1D). In contrast, circulating redox status showed a divergent pattern, with significant post-race reductions in plasma ROS (*p* = 0.0043; Fig. 1E) and TAC (*p* = 0.0041; Fig. 1F).

### Microvascular hemorheology and hemostasis

**Fig. 2:**
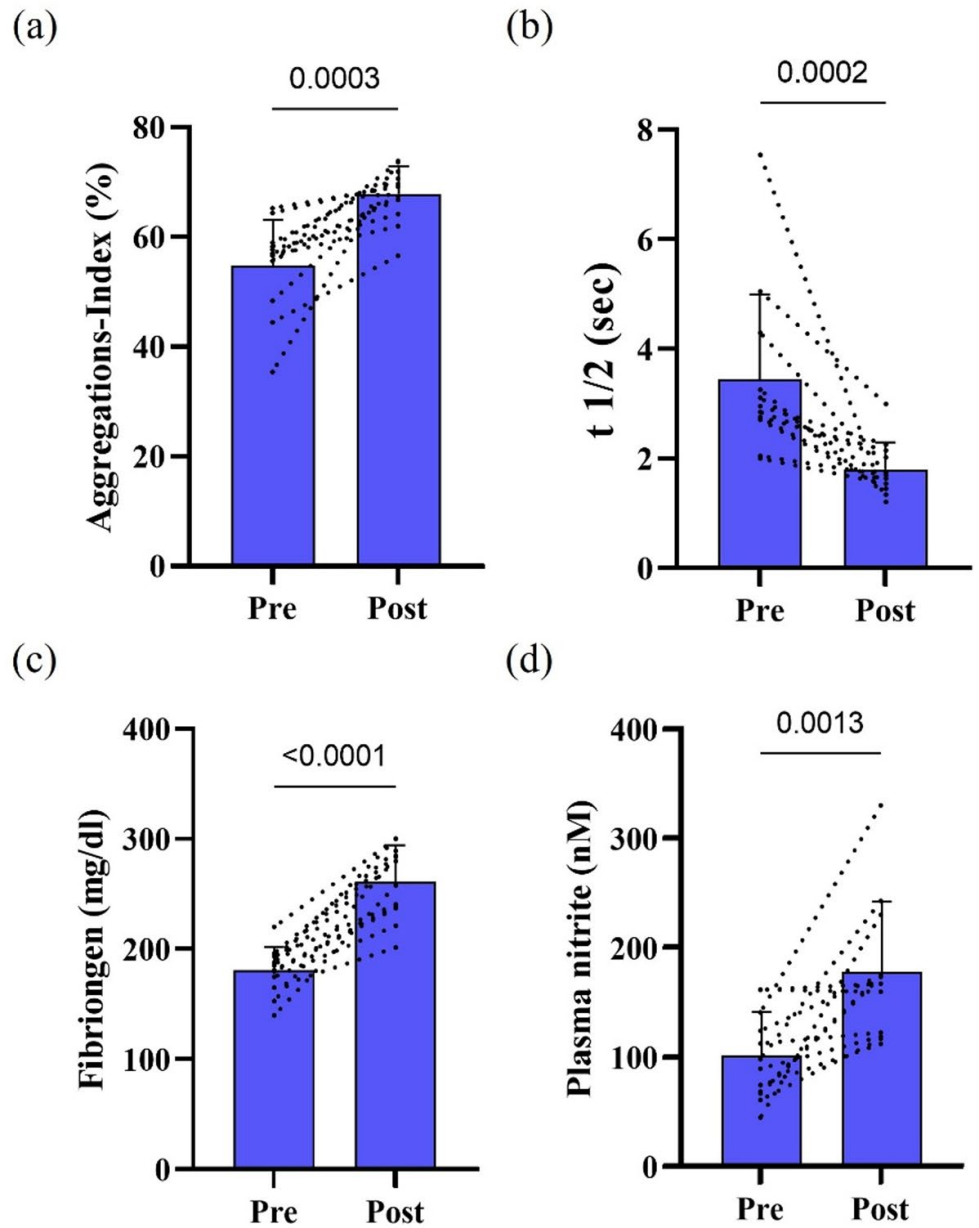
Peri-race changes in RBC aggregation and fibrinogen following an ultramarathon. (**A**) RBC aggregation index increased and (**B**) aggregation half-time (t½) decreased post-race. (**C**) Plasma fibrinogen concentrations were higher post-race, as well as (**D**) plasma nitrite concentrations. Exact p-values are reported within each panel. Individual pre–post trajectories are shown as dashed lines, with bars representing mean ± SD (*n* = 12).

Post-race measurements revealed a significant increase in RBC aggregation index (*p* = 0.0003; Fig. 2A), accompanied by a significant reduction in aggregation half-time (t1/2; *p* = 0.0002; Fig. 2B). Fibrinogen concentrations were significantly elevated after the ultramarathon (*p* < 0.0001; Fig. 2C) as well as plasma nitrite concentrations (*p* = 0.0013; Fig. 2D).

### Macrovascular hemodynamics

Vascular parameters were successfully obtained in *n* = 11 participants, as valid measurements could not be recorded in one participant. Post-race macrovascular responses are presented in Table 1. Outcomes were characterized by a marked increase in heart rate (*p* < 0.0001), accompanied by a reduction in brachial diastolic (*p* = 0.045) and mean arterial pressure (*p* = 0.043), while brachial systolic (*p* = 0.091) and pulse pressure (*p* = 0.382) remained unchanged. Wave reflection indices at the brachial level decreased (AIx: *p* = 0.034), whereas heart rate–standardized AIx75 did not change (*p* = 0.104). Central (aortic) hemodynamic indices, including systolic blood pressure (*p* = 0.136) and PP (*p* = 0.245) as well as aortic AIx (*p* = 0.141), remained unchanged from pre- to post-race. In contrast, DRA increased post-race (*p* = 0.020)), while PWV was preserved (*p* = 0.257).

**Table 1:** Macrovascular hemodynamic parameters before (Pre) and after (Post) the ultramarathon with corresponding p-value. Data are mean ± SD of *n* = 11.

	Pre	Post	*p*-value
HR (bpm)	59.5 ± 8.6	74.7 ± 8.4	< 0.0001
SBP brachial (mmHg)	133.8 ± 12.2	127.7 ± 18.4	0.091
DBP brachial (mmHg)	77.2 ± 9.5	69.8 ± 11.8	0.045
PP brachial (mmHg)	56.6 ± 12.4	57.6 ± 12.8	0.382
MAP brachial (mmHg)	96.1 ± 8.7	89.1 ± 12.9	0.043
AIx brachial (%)	-13.5 ± 29.0	-30.8 ± 21.6	0.034
AIx75 (calculated; %)	-21.2 ± 28.7	-30.7 ± 21.3	0.104
SBP aortic (mmHg)	129.4 ± 17.4	124.9 ± 15.3	0.136
PP aortic (mmHg)	52.2 ± 15.3	52.7 ± 14.3	0.245
AIx aortic (%)	26.7 ± 11.6	22.0 ± 10.9	0.141
DRA	54.6 ± 19.2	71.7 ± 21.7	0.020
PWV (m/s)	9.1 ± 1.3	9.5 ± 1.3	0.257

### Correlation analyses

Spearman correlation analyses were performed to exploratively assess associations between inflammatory, hemorheological, and vascular responses following the ultramarathon. Changes in RBC aggregation were positively associated with changes in fibrinogen concentrations (*r* = 0.636, *p* = 0.015). In contrast, changes in IL-6 and fibrinogen showed an inverse association (*r* = − 0.599, *p* = 0.020). In addition, a moderate trend-level association was observed between changes in plasma nitrite and brachial AIx (*r* = 0.526, *p* = 0.073), although statistical significance was not reached.

## Discussion

Complementary to previous work from our group focusing on RBC deformability and intracellular RBC signaling mechanisms^17^, the present exploratory study specifically addressed the association between inflammatory activation, RBC aggregation/hemostasis, endothelial nitric oxide bioavailability reflected by plasma nitrite levels, and macrovascular hemodynamics following a 230-km non-stop ultramarathon. The main findings were: (i) a pronounced systemic inflammatory response characterized by increases in IL-6, IL-10, CRP, and WBC count, (ii) concomitant alterations in microvascular hemorheology including increased RBC aggregation and fibrinogen concentrations, (iii) elevated plasma nitrite concentrations indicating altered NO signaling, and (iv) largely preserved central arterial stiffness despite changes in peripheral hemodynamics and wave reflection indices. Importantly, these findings indicate that inflammatory activation, hemorheological alterations, and vascular responses are closely interconnected during extreme endurance exercise.

### Systemic and inflammatory and redox stress responses

The ultramarathon elicited a robust inflammatory response, as reflected by significant increases in IL-6, CRP, and WBC count.

IL-6 is primarily secreted during exercise by contracting skeletal muscle as a myokine and functions as both a pro-inflammatory cytokine and a metabolic regulator, linking muscular workload to systemic immune signaling^26^. Increased IL-6 concentrations after ultramarathon running have been consistently reported, with studies reporting increases ranging from several-fold to more than 100-fold depending on race duration and physiological strain^1,27–29^, and the present findings are in line with these observations. Individual IL-6 responses showed substantial heterogeneity, with increases ranging from 32 to 301 pg/mL. Interestingly, the magnitude of IL-6 elevation was not related to running time, suggesting that IL-6 responses may primarily reflect internal physiological stress rather than external performance outcomes. Previous work indicates that muscle glycogen availability is an important determinant of exercise-induced IL-6 release^30^. Under conditions of low glycogen availability, contracting skeletal muscle increases IL-6 secretion, which subsequently stimulates hepatic glucose production and mobilization of alternative substrates to maintain energy homeostasis^31,32^. Data obtained from the same cohort demonstrated increased plasma glucagon concentrations and markers of muscle damage after the ultramarathon^3^, supporting the notion that metabolic stress contributes to the observed cytokine response. While glycogen depletion is considered an important metabolic stimulus for exercise-induced IL-6 release, several additional mechanisms – including contraction-related signaling, muscle damage, and stress-mediated pathways – have also been proposed^33^. Nevertheless, the metabolic and muscular stress observed in the present cohort is consistent with a multifactorial stimulation of IL-6 release during extreme endurance exercise.

The increase in CRP observed in the present study further supports the presence of a systemic inflammatory reaction. IL-6 stimulates hepatic synthesis of CRP as part of the acute-phase response^34^, and increased CRP levels have been consistently documented following ultra-endurance events^1^. In parallel, IL-10 concentrations increased significantly after the race. IL-10 acts as an anti-inflammatory cytokine that limits excessive immune activation and promotes resolution of inflammation. Its increase after strenuous endurance exercise is closely linked to IL-6 signaling, as IL-6 stimulates counter-regulatory anti-inflammatory pathways including IL-10^33,35^.

In contrast to the marked inflammatory activation observed after the ultramarathon, circulating redox markers showed a reduction in both plasma ROS/RNS and TAC. At first glance, this observation appears inconsistent with the widely reported increase in oxidative stress following prolonged endurance exercise^36^. However, circulating redox markers are strongly influenced by sampling timing and compartmental distribution. During prolonged exercise, reactive species are primarily generated within contracting skeletal muscle and inflammatory cells rather than directly in plasma^37^. Consequently, systemic plasma measurements may not directly reflect intracellular oxidative processes. One plausible explanation is that circulating ROS may have been rapidly scavenged by antioxidant systems during the recovery phase following extreme exercise. The simultaneous reduction in plasma TAC observed in the present study supports the interpretation that antioxidant reserves were utilized in response to exercise-induced oxidative processes. Similar patterns have been described in endurance exercise studies reporting increased oxidative damage markers despite reduced circulating antioxidant capacity^17,38,39^. In addition, prolonged exercise promotes redistribution and cellular uptake of reactive species and antioxidants between plasma, RBC, and tissues, which may lead to transient reductions in measurable circulating ROS despite ongoing intracellular oxidative activity^17,37,40^.

Accordingly, the observed decrease in free plasma ROS/RNS does not necessarily indicate reduced overall oxidative stress but rather compartment-specific alterations in circulating redox status following the ultramarathon. This interpretation is supported by our previous findings from the same cohort demonstrating similarly reduced free ROS/RNS levels within RBC despite pronounced systemic physiological stress^17^, suggesting a compartmentalized redox response rather than a uniform reduction in oxidative burden. Together, these findings indicate a coordinated inflammatory response accompanied by an acute shift in circulating redox status.

### Hemorheological and hemostatic responses

In addition to inflammatory activation, the ultramarathon was associated with hemorheological alterations that may influence microcirculatory flow characteristics. RBC aggregation increased markedly, accompanied by a reduction in aggregation half-time and a significant elevation in plasma fibrinogen concentrations, indicating enhanced and more rapid aggregate formation. Moreover, changes in RBC aggregation were positively correlated with changes in fibrinogen, suggesting a functional link between acute-phase activation and post-race hemorheological changes. Importantly, hematocrit – which also influences RBC aggregation – remained unchanged in the present cohort, as reported previously^17^. Also, body weight as well as plasma and blood volume remained stable post-race and fluid intake was considered sufficient^17^, indicating that the observed alterations were not driven by changes in cell concentration.

These observations indicate that exercise-induced increases in fibrinogen, which may partly reflect IL-6–mediated acute-phase synthesis in the liver^34^, contribute to altered RBC aggregation dynamics. IL-6 is rapidly released from contracting skeletal muscle during exercise and typically peaks immediately post-exercise^33,41^, whereas fibrinogen represents a downstream acute-phase protein synthesized in the liver with comparatively delayed kinetics^34^. Consequently, fibrinogen concentrations may continue to increase during the early recovery period even when peak IL-6 responses have already occurred. In line with these differing temporal dynamics, changes in IL-6 and fibrinogen were inversely correlated in the present study, suggesting that the immediate post-race time point likely captured distinct phases of the inflammatory and acute-phase response. Beyond its role as an acute-phase protein, fibrinogen is a key determinant of RBC aggregation because it promotes reversible bridging interactions between RBC, facilitating rouleaux formation particularly under low shear conditions and thereby increasing blood viscosity that may modify microvascular flow properties^7^. In the context of acute inflammatory activation, elevated fibrinogen concentrations may enhance these cell–cell interactions, promoting faster aggregate formation and increased aggregation. Previous work has demonstrated that hemorheological parameters such as RBC aggregation can change after prolonged endurance exercise, although direct evidence following ultramarathons remains limited^8,42^. The present findings extend these observations by demonstrating concomitant increases in fibrinogen and RBC aggregation following a 230-km non-stop ultramarathon, including a significant association between changes in both parameters. Taken together, these findings suggest that inflammation-driven acute-phase responses may transiently increase fibrinogen-dependent RBC aggregation, thereby suggesting a potential influence on microvascular rheology during the immediate post-race period.

These acute changes support the notion that post-race fibrinogen dynamics reflect an integrated immune–hemostatic response rather than an isolated coagulation phenomenon^1^. Alterations in hemorheology may additionally influence microvascular flow characteristics and peripheral vascular regulation, thereby linking inflammatory activation to vascular function, although microvascular function was not directly assessed in the present study.

### Endothelial and macrovascular response

Another notable finding of the present study was the increase in plasma nitrite concentrations following the ultramarathon. Plasma nitrite is widely used as a surrogate marker of systemic NO bioavailability^43,44^. NO is a central regulator of vascular tone and endothelial function, and prolonged endurance exercise is known to stimulate NO production through increased shear stress and endothelial activation^45,46^. The observed increase in plasma nitrite may therefore reflect enhanced systemic NO bioavailability which may be associated with alterations in vascular NO metabolism following the ultramarathon. Complementary analyses from the same cohort additionally demonstrated increased RBC nitrite levels and enhanced RBC-NO synthase (RBC-NOS) activation following the ultramarathon^17^. Given the close mechanistic and regulatory similarities between RBC-NOS and endothelial NOS (eNOS) pathways^47,48^, these findings are consistent with the possibility that the elevated plasma nitrite concentrations observed in the present study may, at least in part, reflect increased exercise-induced eNOS-related NO production. Mechanistically, increased NO availability promotes vasodilation within resistance vessels of the peripheral circulation, thereby reducing systemic vascular resistance^45^. Such vasodilatory adjustments provide a plausible mechanistic link to the hemodynamic changes observed in the present study. Supporting this interpretation, exploratory correlation analyses revealed a moderate association between changes in plasma nitrite and brachial AIx, although statistical significance was not reached. Given the sensitivity of AIx to peripheral vascular tone and wave reflection characteristics, this trend-level finding may reflect NO-related contributions to post-race vascular adjustments following the ultramarathon. Specifically, post-race responses were characterized by a significant increase in heart rate accompanied by reductions in brachial diastolic and mean arterial pressure. The elevated heart rate likely reflects a compensatory response to reduced peripheral resistance aimed at maintaining cardiac output in the presence of sustained vasodilation. Such a pattern is consistent with the well-described phenomenon of post-exercise hypotension, which has been attributed primarily to sustained peripheral vasodilation and reduced systemic vascular resistance after prolonged endurance exercise^45,49^. Importantly, these measurements were intentionally obtained during the immediate post-race recovery phase and should therefore be interpreted as reflecting acute circulatory adjustments rather than stabilized resting vascular function.

Changes in peripheral vascular tone and resistance also influence the characteristics of arterial pressure wave propagation and reflection^50^. Pulse wave analysis therefore provides an integrative approach to assess how microvascular and endothelial changes translate into macrovascular hemodynamics. In the present study, wave reflection indices at the brachial level decreased, as indicated by the reduction in AIx values. However, heart-rate–standardized AIx75 remained unchanged, suggesting that the apparent reduction in AIx was primarily driven by the elevated post-race heart rate rather than reflecting a substantial change in wave reflection properties. Given the known dependence of AIx on heart rate and peripheral vascular tone^16^, these findings are more consistent with transient functional hemodynamic adjustments following the ultramarathon than with structural changes in arterial properties. Although prolonged mechanical and metabolic stress may theoretically induce transient alterations within the vascular extracellular matrix^45^, the present findings are more consistent with functional adjustments of vascular tone.

Central hemodynamic indices, including aortic systolic pressure, aortic pulse pressure, and aortic AIx, remained largely unchanged, and central arterial stiffness assessed by PWV did not change significantly. Further, DRA – and thus perfusion of the left coronary – increased which may indicate altered diastolic filling dynamics^51^.

This suggests that despite pronounced systemic inflammatory activation and rheological alterations – including marked increases in IL-6, CRP, and fibrinogen observed in the present cohort – the mechanical properties of the central arterial system were largely preserved in the immediate post-race period, indicating that the acute cardiovascular response may be dominated by functional adjustments in peripheral vascular tone rather than structural changes in central arterial stiffness. This interpretation is consistent with previous ultramarathon studies demonstrating heterogeneous macrovascular responses. For example, Koutlianos^12^ observed increased PWV following a 246-km ultramarathon, with CRP correlating with downstream vascular resistance, suggesting an inflammation-related stiffening response under certain race conditions. In contrast, Babcock^11^ reported unchanged PWV after a 100-mile ultramarathon despite pronounced post-race hypotension and marked reductions in AIx and central hemodynamic indices. Such dissociation indicates that central arterial stiffness and wave reflection may respond independently following ultra-endurance exercise. However, direct comparison between studies remains challenging because vascular assessments in ultramarathon research are performed under substantially different post-race conditions and recovery time points, ranging from immediate post-finish measurements to more stabilized resting assessments. Additionally, investigations of shorter ultramarathons have demonstrated complex, time-dependent vascular responses across multiple domains, including changes in wave reflection indices and microvascular function^13,14^.

The present findings therefore support the concept that acute peripheral vascular adjustments associated with inflammatory and endothelial signaling may occur without substantial alterations in central arterial stiffness following extreme endurance exercise.

Importantly, the concomitant increase in IL-6 and CRP, fibrinogen-related hemorheological alterations, and enhanced NO signaling suggests that inflammatory and endothelial processes may contribute to peripheral vascular regulation during the immediate post-race period and may be associated with concurrent macrovascular hemodynamic responses.

Accordingly, extreme endurance exercise appears to induce pronounced inflammatory, hemorheological, and endothelial responses while central arterial stiffness remains largely preserved in the immediate post-race period. Strategies aimed at minimizing excessive physiological stress during and after prolonged endurance exercise – such as adequate metabolic fueling and structured post-race recovery^52,53^– may therefore help mitigate downstream hemorheological and cardiovascular strain.

### Strengths and limitations

A major strength of the present study is the parallel assessment of inflammatory, microvascular, endothelial, and macrovascular parameters within the same cohort of athletes participating in an exceptionally long non-stop ultramarathon. This multidimensional approach enabled a comprehensive evaluation of how inflammatory activation, hemorheological alterations, and vascular responses interact during extreme endurance exercise. Several limitations should also be considered. The study included a relatively small sample size, which reflects the logistical challenges of conducting physiological measurements in the context of a 230-km ultramarathon and the study cohort represented a highly selected group of very experienced ultramarathon runners (> 60 completed ultramarathons), which may limit the generalizability of the findings to the broader ultramarathon population. Due to the exploratory pilot nature and relatively small sample size of the present study, non-significant findings—particularly regarding central hemodynamic parameters—should be interpreted cautiously, as smaller physiological effects may have remained undetected. The measurements were performed immediately after the race, and therefore the temporal dynamics of recovery could not be assessed. Macrovascular parameters were derived from oscillometric pulse wave analysis rather than being directly assessed using invasive reference-standard techniques, although the applied device has previously been validated against established methods^20–22^. The observational design precludes causal inference regarding the mechanistic interactions between inflammatory, hemorheological, and vascular responses. Finally, environmental factors such as temperature, hydration status, and nutritional intake during the race could not be fully standardized but individual energy and fluid intake have been previously described^3^.

### Conclusion

The present study demonstrates that a 230-km non-stop ultramarathon elicits a pronounced systemic inflammatory response accompanied by marked hemorheological alterations including increased RBC aggregation and fibrinogen concentrations. These responses occurred alongside modest changes in peripheral hemodynamics, whereas central arterial stiffness remained largely preserved in the immediate post-race period.

Together, the present findings indicate that extreme endurance exercise is associated with parallel inflammatory, hemorheological, endothelial, and vascular responses that may contribute to acute alterations in peripheral vascular regulation rather than structural changes in central arterial properties. However, given the exploratory design and limited sample size, definitive mechanistic interactions between these systems cannot be concluded. Future studies using larger cohorts and mechanistic or multivariate approaches are warranted to further clarify these interrelationships, investigate the temporal recovery of these responses, and examine potential strategies to mitigate inflammatory and hemorheological stress following extreme endurance exercise.

## Data Availability

The data that support the ﬁndings of this study are available upon reasonable request from the corresponding author. The data are not publicly available due to privacy or ethical restrictions.

## Abbreviations

Aix: Augmentation index
AI %: Aggregation index
CRP: C-reactive protein
DBP: Diastolic blood pressure
DRA: Diastolic reflection area
HR: Heart rate
IL: Interleukin
MAP: Mean arterial pressure
NO: Nitric oxide
NO_2_^−^: Nitrite
PWV: Pulse wave velocity
RBC: Red blood cells
ROS: Reactive oxygen species
SBP: Systolic blood pressure
PP: Pulse pressure
TAC: Total antioxidant capacity
WBC: White blood cells
